# Extracorporeal membrane oxygenation for systemic lupus erythematosus: An ELSO registry analysis

**DOI:** 10.1177/02676591251346069

**Published:** 2025-05-26

**Authors:** Maria Pereira, Danielle Guffey, Katherine Doane, Eyal Muscal, Carla Levin, Peter Rycus, Marc Anders, Andrea Ontaneda

**Affiliations:** 1 Division of Pediatric Rheumatology, 3989 Baylor College of Medicine, Houston, TX, USA; 2 Institute for Clinical & Translational Research, 3989 Baylor College of Medicine, Houston, TX, USA; 3 Division of Pediatric Critical Care, 3989 Baylor College of Medicine, Houston, TX, USA; 4 Department of Pediatrics, Baylor College of Medicine, Houston, TX, USA; 5 634749 The Extracorporeal Life Support Organization, Ann Arbor, MI, USA

**Keywords:** Systemic Lupus Erythematosus, lupus, extracorporeal membrane oxygenation, extracorporeal life support, ELSO registry

## Abstract

The success of extracorporeal membrane oxygenation (ECMO) in treating Systemic Lupus Erythematosus (SLE) and the risk factors associated with mortality remain uncertain.

*Methods:* We describe the survival outcomes at discharge of the largest SLE cohort on ECMO support. We performed a retrospective cohort study of the Extracorporeal Life Support Organization registry database from 2012 to 2022. Pediatric and adult survivor groups were analyzed using descriptive statistics for the primary study outcome of survival to hospital discharge. Risk predictors for survival were determined by logistic regression.

*Results:* We included 48 children and 368 adults with SLE. Overall, 198 patients (54%) survived to hospital discharge, with a survival of 52% and 47% of pediatric and adult patients, respectively.

*Conclusion:* We conclude that ECMO can be considered a life supporting strategy in pediatric and adult SLE patients.

## Introduction

Systemic Lupus Erythematosus (SLE) is a clinically heterogeneous multisystemic autoimmune disorder with potentially life-threatening manifestations. Global epidemiology reports a premature mortality ratio to be as 2 to 3 times higher than that of the general population, especially in young non-white women.^1^ The leading cause of death in SLE is related to cardiovascular complications due to atherosclerosis, stroke, myocardial infarction, and heart failure.^2,3^ Infections account for most of the mortality in hospitalized children and adolescents with SLE, with an increased risk in those with severe kidney involvement.^4^ Diffuse alveolar hemorrhage, although rare, is a devastating pulmonary complication of SLE with high mortality affecting all age groups.^5,6^ The median ICU mortality rate of adult SLE patients remains as high as 29.6% (IQR 24-47) but has decreased from 46.7% (IQR 31.8-57) before 2010.^7^

Extracorporeal membrane oxygenation (ECMO) has supported patients with cardiac, respiratory, or cardiorespiratory failure since the 1970s.^8^ While relatively contraindicated in the past, ECMO is being increasingly utilized to support patients with immune dysfunction. Recently published studies on acute respiratory distress syndrome (ARDS) supported with ECMO report that 19-31% of patients are immunocompromised.^9,10^ The utility of ECMO in SLE and the risk factors associated with mortality in this population remain unclear. Our study aims to describe the use and survival outcomes of ECMO in SLE patients.

## Methods

### Study design

Retrospective cohort study of the Extracorporeal Life Support Organization (ELSO) Registry database from 2012 to 2022 including patients of all ages who had International Classification of Diseases Ninth Revision (ICD9) and Tenth Revision (ICD10) codes consistent with SLE. Data collected included basic demographic information, comorbid diagnosis, pre-ECMO respiratory and hemodynamic support parameters, ECMO indication, cannulation method, ECMO mode, duration of ECMO, procedures performed, and ECMO complications. The decision to use ECMO was made independently at each center. Comorbidities were entered via ICD9/10 coding. Pulmonary involvement was defined using codes for respiratory failure, acute respiratory distress syndrome, pulmonary hemorrhage, hemoptysis, pulmonary hypertension, and pneumothorax. Renal involvement was defined using codes for acute kidney injury, chronic kidney disease, end-stage renal disease, glomerulonephritis. Cardiac disease was defined using codes for pericarditis, pericardial effusion, endocarditis, myocarditis, and arrhythmias. Procedures defined by ICD9/10 coding included renal replacement therapy, dialysis, bronchoscopy, thoracocentesis, chest tube, tracheostomy, and plasmapheresis (Appendix, supplemental content on ICD9/10 and CPT codes).^11^ ECMO complications were predefined by ELSO definitions and listed by categories.^12^ Patient and mechanical complications were determined by their need for intervention and associated with the ECMO run. Data on lupus severity or immunosuppression therapy was not included as it is not available in the registry. This study was approved by the ELSO Registry Scientific Oversight Committee and granted an exemption by the Institutional Review Board of Baylor College of Medicine.

### Statistical analysis

Descriptive analysis for demographics, ECMO parameters, and ECMO complications were used for the primary study outcome of survival to hospital discharge. The analyses were performed for the overall cohort and the pediatric (30 days to <18 years old) and adult subgroups. The number of patients with documented data by variable is indicated in the tables.

Baseline characteristics are presented as mean with standard deviation, median with 25^th^ and 75^th^ percentiles or frequency with percentage. We utilized Wilcoxon rank sum test, Fisher’s exact test, or chi-square test to compare characteristics by survival to hospital discharge. We performed logistic regression to determine associations between risk predictors and survival to hospital discharge. The statistical analysis was completed using Stata v 18 (StataCorp, College Station, TX, USA).

## Results

During the study period, 48 children, median (IQR) age 15.1 (12.3,16.8) years, and 368 adults, median (IQR) age 40.4 (29.8, 51.8) years, were identified from the ELSO registry with ICD9 or ICD10 codes for SLE who had received ECMO support. Overall, 198 patients (48%) survived to hospital discharge, with survival of 52% and 47% of pediatric and adult patients, respectively. Most adult (82.6%) and pediatric (85.4%) patients were female. In children, Black was the predominant race in both non-survivors (34.8%) and survivors (32%). 50 percent of adults and pediatric patients had respiratory failure before ECMO initiation.

Acute kidney injury was present pre-ECMO in 32% of adult patients, and 37% of pediatric patients. Preexistent bacterial infection, before ECMO support, was identified in 23.1% of the adult non-survivor group versus 13.3% in the adult survivor group (*p* = .022). Pre-ECMO cardiac arrest was observed in 33% of pediatric patients (30% non-survivor group, 36% survivor group, *p* = .765). In the adult population, 26% had pre-ECMO cardiac arrest (30% non-survivor group, 20% survivor group, *p* = .031), and was associated with decreased survival to hospital discharge (OR 0.58 [0.36 – 0.95], *p* = .029). Adult patients in the survivor group had a median (IQR) arterial oxygen partial pressure (PaO2) of 76 mmHg (59, 130) versus 65 mmHg (50, 107.5) in the non-survivor group (*p* = .007), and oxygen saturation of 94% (88, 99) versus 90% (82, 94) (*p* = .003). None of these variables were significant in children. Tables 1 and 2 show the demographic and pre-ECMO support survival characteristics by cohort.

**Table 1.** Demographic characteristics.

	Pediatric (*n* = 48)			Adult (*n* = 368)		
Characteristics	Non-survivors	Survivors		Non-survivors	Survivors	
No. (%) or median (IQR)	(*n* = 23)	(*n* = 25)	*p*	(*n* = 195)	(*n* = 173)	*p*
Age (year)	15.5 (13.1,16.9)	14.1 (12.1,16.7)	0.227	42.1 (31.9,54.1)	37.6 (27.6,48.3)	0.010
Female	20 (87.0)	21 (84.0)	1.000	165 (84.6)	139 (80.3)	0.505
Race/Ethnicity			0.563			0.615
Asian	3 (13.0)	1 (4.0)		27 (13.8)	14 (8.1)	
Black	8 (34.8)	8 (32.0)		53 (27.2)	53 (30.6)	
Hispanic	3 (13.0)	7 (28.0)		22 (11.3)	25 (14.5)	
White	3 (13.0)	5 (20.0)		64 (32.8)	55 (31.8)	
Other	6 (26.0)	3 (12.0)		20 (10.2)	20 (11.5)	
Comorbidities						
Acute kidney disease	6 (26.1)	12 (48.0)	0.248	66 (33.8)	52 (30.1)	0.502
Chronic kidney disease	0. (0.0)	2 (8.0)	0.490	32 (16.4)	24 (13.9)	0.562
Arrythmia	1 (4.3)	1 (4.0)	1.000	26 (13.3)	17 (9.8)	0.332
Pulmonary hemorrhage	1 (4.3)	2 (8.0)	1.000	10 (5.1)	15 (8.7)	0.215
Respiratory failure	9 (39.1)	15 (60.0)	0.248	101 (51.8)	92 (53.2)	0.835
ARDS	2 (8.7)	4 (16.0)	0.068	34 (17.4)	36 (20.8)	0.427
Pneumothorax	1 (4.3)	1. (4.0)	1.000	12 (6.2)	11 (6.4)	1.000
Pneumonia	1 (4.3)	1 (4.0)	1.000	27 (13.8)	29 (16.8)	0.332
Pre existent infection	10 (43.5)	8 (32.0)	0.552	57 (29.2)	33 (19.1)	0.029
Bacterial	8 (34.8)	6 (24.0)	0.529	45 (23.1)	23 (13.3)	0.022
Viral	2 (8.7)	3 (12)	1.000	14 (7.2)	11 (6.4)	0.837
Fungal	3 (13)	0 (0)	0.102	13 (6.7)	6 (3.5)	0.238
Shock	3 (13.0)	5 (20.0)	0.703	45 (23.1)	31 (17.9)	0.247
Sepsis	8 (34.8)	4 (16.0)	0.187	41 (21.0)	29 (16.8)	0.352

**Table 2.** Pre-ECMO support.

	Pediatric (*n* = 48)			Adult (*n* = 368)		
Variable	Non-survivors	Survivors		Non-survivors	Survivors	
No. (%) or median (IQR)	(*n* = 23)	(*n* = 25)	*p*	(*n* = 195)	(*n* = 173)	*p*
Medications						
Corticosteroids	5 (21.7)	7 (28.0)	0.743	67 (34.4)	51 (29.5)	0.371
Inotropic	21 (91.3)	16 (64.0)	0.039	124 (63.6)	104 (60.1)	0.520
Neuromuscular blockade	13 (56.5)	14 (56.0)	1.000	60 (30.8)	69 (39.9)	0.080
Nitric oxide	8 (34.8)	8 (32.0)	1.000	28 (14.4)	21 (12.1)	0.543
Vasodilator	2 (8.7)	2 (8.0)	1.000	9 (4.6)	9 (5.2)	0.813
Betablocker	0 (0.0)	0 (0.0)	NS	1 (0.5)	3 (1.7)	0.346
Prostaglandins	0 (0.0)	0 (0.0)	NS	18 (9.2)	23 (13.3)	0.247
Ventricular assist device	0 (0.0)	0 (0.0)	NS	3 (1.5)	2 (1.2)	1.000
High flow nasal cannula	3 (13.0)	1 (4.0)	0.338	2 (1.0)	2 (1.2)	1.000
Mechanical ventilation type			0.592			0.002
Conventional	19 (82.6)	16 (72.7)		155 (95.7)	122 (84.1)	
High frequency oscillator	1 (4.3)	3 (13.6)		0	0	
No ventilator	3 (13.0)	3 (13.6)		5 (3.1)	18 (12.4)	
Other	0 (0.0)	0 (0.0)		2 (1.2)	5 (3.4)	
Intubation to ECMO time (hrs)	13 (5.0,53.0)	13 (3.0,52.0)	0.974	13.5 (4.0,68.0)	11 (4.0,52.0)	0.646
PEEP (cmH_2_0)	12 (8.0,15.0	12 (10.0,15.0)	0.821	10 (6.0,12.0)	12 (7.5,14.5)	0.017
MAP (cmH_2_0)	20 (15.0,28.0)	22 (16.5,25.0)	0.604	20 (14.0,23.0)	20 (15.0,25.0)	0.403
FiO2 (%)	100 (100.0.100.0)	100 (100.0.100.0)	0.775	100 (100.0.100.0)	100 (80.0.100.0)	0.095
PaO2 (mmHg)	72.1 (46.0, 109.0	71 (56.0, 102.0)	0.787	65 (50.0, 107.5)	76 (59.0, 130.0)	0.007
PaCO2 (mmHg)	47 (37.0, 62.0)	44.5 (32.1, 67.0)	0.947	49.8 (39.9, 66.9)	45 (36.0, 65.0)	0.179
SpO2 (%)	98 (81.0, 99.0)	91 (82.0, 97.0)	0.932	90 (82.0, 94.0)	94 (88.0, 99.0)	0.003
PIP (cmH_2_0)	32 (24.0, 44.0)	31.5 (23.5, 38.0)	0.942	33 (26.0, 38.0)	34 (27.0, 38.0)	0.734
SBP (mmHg)	99 (64.0, 134.0)	93.5 (48.0, 67.0)	0.605	93 (80.0, 116.0)	107 (91.0, 124.0)	<0.001
DBP (mmHg)	52.5 (37.0, 69.0)	62 (48.0, 67.0)	0.409	54 (45.0, 64.0)	60.5 (50.0, 74.0)	0.001
Mean BP (mmHg)	73.5 (53.0, 89.5)	71 (61.0, 86.0)	0.958	67 (57.0, 80.0)	74 (62.0, 90.0)	0.001
Cardiac arrest procedures	7/16 (30)	9/16 (36)	0.765	59/94 (26)	35/94 (20)	0.031
Bronchoscopy	0	1 (4)	1.000	10 (5.1)	9 (5.2)	1.000
Thoracocentesis	0	1 (4)	1.000	2 (1.0)	2 (1.2)	1.000
Tracheostomy	0	1 (4)	1.000	2 (1.0)	2 (1.2)	1.000
Plasmapheresis	1 (4.3)	2 (8.0)	1.000	2 (1.0)	2 (1.2)	1.000
Renal replacement	1 (4.3)	0 (0.0)	0.479	5 (2.6)	3 (1.7)	0.728

PEEP: Positive end-expiratory pressure; MAP: Mean airway pressure; FIO2: Fraction of inspired oxygen; PaO2: Partial pressure of oxygen; PaCo2: Partial pressure of carbon dioxide; SpO2: oxygen saturation; PIP: Peak inspiratory pressure; SBP: Systolic blood pressure; DBP: Diastolic blood pressure; Mean BP: Mean blood pressure.

The indication for ECMO support was 50% respiratory for both the pediatric and adult cohorts, and 40% and 31% cardiac support for adults and pediatrics, respectively. ECMO-cardiopulmonary resuscitation (ECPR) was the support type in 9% of adults and 19% of pediatrics. The median (IQR) duration for ECMO run was 149 hours (76, 228) in pediatrics and 155 hours (73, 350) in adults. Veno-arterial (VA) ECMO mode was utilized in 60% of the pediatric cohort, whereas in the adult population VA mode was utilized in 47.6%, and Veno-venous (VV) ECMO mode in 49.5%. (Table 3 shows ECMO support by survival status at discharge).

**Table 3.** ECMO support.

	Pediatric (*n* = 48)			Adult (*n* = 368)		
Variable	Non-survivors	Survivors		Non-survivors	Survivors	
No. (%) or median (IQR)	*n* = 23	(*n* = 25)	*p*	(*n* = 195)	(*n* = 173)	*p*
Support type			0.163			<0.001
Pulmonary	14 (60.9)	10 (40.0)		89 (45.6)	103 (59.5)	
Cardiac	4 (17.4	11 (44.0)		79 (40.5)	65 (37.6)	
ECPR	5 (21.7)	4 (16.0)		27 (13.8)	103 (59.5)	
ECMO mode			0.552			0.023
VV	10 (43.5)	8 (32.0)		84 (43.1)	98 (56.6)	
VA	13 (56.5)	16 (64.0)		104 (53.3)	71 (41.0)	
VP	0 (0.00)	1 (4.0)		0 (0.0)	1 (0.6)	
VVA	0 (0.00)	0 (0.00)		7 (3.6)	3 (1.7)	
Conversion	2 (8.7)	1 (4.0)		14 (7.2)	13 (7.5)	
Duration (hours)	139 (39.0, 303.0)	158 (94.0, 197.0)	0.836	157.5 (51.0, 355.0)	153 (93.0, 348.0)	0.257
Procedures						
Bronchoscopy	4 (17.4	7 (28)	0.499	42 (21.5)	61 (35.3)	0.004
Tracheostomy	3 (13)	1 (4)	0.338	13 (6.7)	8 (4.6)	0.501
Thoracocentesis	3 (13)	1 (4)	0.338	13 (6.7)	8 (4.6)	0.501
Plasmapheresis	4 (17.4) 7 (28.0)		0.499	4 (2.1)	8 (4.6)	0.240
Renal replacement	4 (17.4) 3 (12.0)		0.696	17 (8.7)	8 (4.6)	0.147

ECPR: Extracorporeal cardiopulmonary resuscitation; VV: Venovenous; VA: Venoarterial; VP: Venopulmonary; VVA: Venovenous to venoarterial.

During ECMO support, both adult and pediatric patients experienced complications (Table 4). Arrhythmia and cardiac arrest were the most common cardiovascular complications in the adult non-survivor group, 15.4% and 11.3%, respectively, while in the survivor group they were present in 5.8% (*p* = .004) and 1.2% (*p* < .001), respectively. CNS infarction was the most common neurological complication in the adult non-survivor group, 8.7% versus 1.2% in the survivor group (*p* = .001). Hemorrhagic complications were more prevalent in the adult non-survivor group, 29.7%, versus 19% in the survivor group (*p* = .021) (Table 4). Adult patients who had cardiovascular, renal, neurologic, and hemorrhagic complications had lower survival to hospital discharge (Table 5). None of these complications in the pediatric cohort affected survival to discharge.

**Table 4.** ECMO complications.

	Pediatric (*n* = 48)			Adult (*n* = 368)		
	Non-survivors	Survivors		Non-survivors	Survivors	
	*n* = 23	(*n* = 25)	*p*	(*n* = 195)	(*n* = 173)	*p*
Cardiovascular	3 (13.0)	1 (4.0)	0.338	54 (27.7)	12 (6.9)	<0.001
Arrhythmia	2 (8.7)	1 (4.0)	0.601	30 (15.4)	10 (5.8)	0.004
Tamponade (blood)	1 (4.3)	0 (0.0)	0.479	5 (2.6)	3 (1.7)	0.728
Tamponade (no blood)	0 (0.0)	0 (0.0)	NS	1 (0.5)	0	1.000
CPR required	1 (4.3)	0 (0.0)	0.479	22 (11.3)	2 (1.2)	<0.001
Neurologic	3 (13.0)	1 (4.0)	0.091	38 (19.5)	5 (2.9)	<0.001
Brain death	0 (0.0)	0 (0.0)	NS	11 (5.6)	0 (0)	0.001
CNS infarction	1 (4.3)	1 (4.0)	1.000	17 (8.7)	2 (1.2)	0.001
CNS ischemia	1 (4.3)	0 (0.0)	0.479	6 (3.1)	0 (0.0)	0.032
Parenchymal hem	0 (0.0)	0 (0.0)	NS	10 (5.1)	2 (1.2)	0.04
Intraventricular hem	2 (8.7)	0 (0.0)	0.224	1 (0.5)	0 (0.0)	1.000
Seizure clinical	0 (0.0)	0 (0.0)	NS	3 (1.5)	1 (0.6)	0.626
Seizure by EEG	0 (0.0)	0 (0.0)	NS	1 (0.5)	0 (0.0)	1.000
Infectious	9 (39.1)	5 (20.0)	0.207	77 (39.5)	61 (35.3)	0.45
Viral	2 (8.7)	3 (12.0)	1.000	7 (3.6)	11 (6.4)	0.238
Bacterial	7 (30.4)	3 (12.0)	0.162	56 (28.7)	42 (24.3)	0.347
Fungal	1 (4.3)	1 (4.0)	1.000	39 (20.0)	34 (19.7)	1.000
Renal	14 (60.9	16 (64.0)	1.000	91 (46.7)	60 (34.7)	0.026
Creatinine 1.5-3 (mg/dL)	5 (21.7)	2 (8.0)	0.237	39 (20.0)	22 (12.7)	0.068
Creatinine >3.0 (mg/dL)	1 (4.3)	1 (4.0)	1 (4.0)	19 (9.7)	8 (4.6)	0.072
Renal replacement	13 (56.5)	16 (64.0)	0.769	76 (39.0)	50 (28.9)	0.048
Hemorrhagic	6 (26.1)	5 (20.0)	0.736	58 (29.7)	33 (19.1)	0.021
Limb ischemia	2 (8.7)	2 (8.0)	1.000	7 (3.6)	2 (1.2	0.181
Mechanical	6 (26.1)	7 (28.0)	1.000	36 (18.5)	26 (15.0)	0.405
Metabolic	5 (21.7)	1 (4.0)	0.091	26 (13.3)	14 (8.1)	0.131
Pulmonary	5 (21.7)	1 (4.0)	0.091	18 (9.2)	16 (9.2)	1

CPR: Cardiopulmonary resuscitation; CNS: Central nervous system.

**Table 5.** Unadjusted logistic regression for survival to discharge in adults.

Variable	Odds ratio (95% CI)	*p*	*n*
Age	0.98 (0.97–0.99)	0.006	368
Pre-existent infection	0.57 (0.35–0.93)	0.003	368
Bacterial	0.51 (0.29–0.89)	0.017	
Viral	0.88 (0.39–2)	0.755	
Fungal	0.5 (0.19–1.35)	0.174	
Pre-ECMO ventilation type		0.006	307
Conventional	1.0 (reference)		
No ventilator	4.57 (1.65–12.67)	0.003	
Other	3.18 (0.61–16.65)	0.172	
PEEP (cmH20)	1.05 (1.00–1.11)	0.033	254
PaO2 (mmHg)	1.00 (1.00–1.00)	0.582	308
SaO2 (%)	1.03 (1.01–1.05)	0.004	265
SpO2 (%)	1.06 (1.02–1.11)	0.007	122
SBP (mmHg)	1.01 (1.01–1.02)	0	312
DBP (mmHg)	1.02 (1.01–1.04)	0.001	311
Mean BP (mmHg)	1.02 (1.01–1.03)	0.001	272
ECMO mode		0.025	367
VA	1.0 (reference)		
VV	1.71 (1.12–2.6)	0.012	
VVA	0.63 (0.16–2.51)	0.51	
Support type		0.001	368
Cardiac	1.0 (reference)		
ECPR	0.23 (0.08–0.62)	0.004	
Pulmonary	1.41 (0.91–2.17)	0.123	
Cardiac arrest	0.58 (0.36–0.95)	0.029	94
Bronchoscopy - on ECMO	1.98 (1.25–3.15)	0.004	368
ECMO complications			
Cardiac	0.19 (0.1–0.38)	<0.011	368
Renal	0.61 (0.4–0.92)	0.02	368
Hemorrhagic	0.56 (0.34–0.91)	0.019	368
Neurologic	0.12 (0.05–0.32)	<0.001	368
Bleeding	0.22 (0.05–1)	0.05	368
Renal replacement therapy	0.64 (0.41–0.99)	0.043	368

PEEP: positive end-expiratory pressure; PaO2: partial pressure of oxygen; Sa02: oxygen saturation.

SBP: systolic blood pressure; DBP: diastolic blood pressure; VA: venoarterial; VV: venovenous.

VVA: venovenous to venoarterial conversion; ECPR: extracorporeal cardiopulmonary resuscitation.

Adult and pediatric patients underwent procedures and therapies pre-ECMO and during ECMO including bronchoscopy, thoracocentesis, tracheostomy, plasmapheresis, and renal replacement therapies (Tables 2 and 3). In the adult survivor group, bronchoscopy during ECMO was performed in 35.3 % versus 21.5% in the non-survivor group (*p* = .004). Only bronchoscopy while on ECMO in the adult population was associated with increased survival to discharge (OR 1.98 [1.25-3.15], *p* = .004) (Table 5). No other procedure or therapy affected survival to discharge.

In the adult population, older age (OR 0.98 [0.97-0.99], *p* = .006), and pre-existent bacterial infection before ECMO initiation (OR 0.51 [0.29-0.89], *p* = .017) were associated with decreased survival to hospital discharge. Adult patients not requiring pre-ECMO invasive mechanical ventilation support had higher odds of survival (OR 4.57 [1.65-12.67], *p* = .003). In the pediatric population, the need for inotropic support pre-ECMO was associated with decreased survival to hospital discharge (OR 0.17 [0.03-0.89], *p* = .036). In the adult population, Veno-venous mode was associated with increased survival to discharge (OR 1.71 [1.12-2.6], *p* = .012), and ECPR was associated with decreased survival to discharge (OR 0.23 [0.08-0.62], *p* = .004) (Table 5).

## Discussion

We describe the survival outcomes at discharge for the largest SLE cohort on ECMO support. In our review of the ELSO registry, there was an overall 48% survival to hospital discharge, with 52% and 47% of pediatric and adult patients, respectively. The Extracorporeal Life Support Organization (ELSO) 2024 International Summary reported an overall adult and pediatric survival to discharge for 59% and 62% pulmonary support, 46% and 55% cardiac support, and 31% and 41% extracorporeal cardiopulmonary resuscitation (ECPR) respectively.^13^

Immunosuppression, infections, clotting, cardiovascular and renal disease associated with SLE may complicate ECMO candidacy decisions in these patients,^5,14^ yet emerging data with favorable survival outcomes support the use of ECMO in the critically ill lupus patient. Recent studies involving adult systemic autoimmune disease patients on ECMO report survival rates of 49% upon hospital discharge,^15,16^ comparable to those of the ELSO registry. Case reports and small case series in pediatric and adults describe variable outcomes^17–30^ (Table 6).

**Table 6.** Summary of Reported SLE Cases supported on ECMO.

Author	Case (age, sex)	Therapies received	ECMO mode	ECMO indication	Complication	Outcome
Shi et al, 2022	43 F	MTP, Rituximab	VA	Cardiac shock	None	Survived
	32 F	MTP	VA	Cardiac shock	RRT, GI bleed	Died
	22 F	MTP, Rituximab	VA	Cardiac shock	Liver failure	Died
Lee et al, 2021	62 F	MTP	VA	Cardiac shock	RRT	Survived
Liu et al, 2020	47 F	MTP, IVIG	VA	Cardiac shock	None	Survived
Smith et al, 2020	25 F	MTP, CPM, IVIG, PLX	VA	Cardiac shock	None	Survived
Wang et al., 2018	3 cases	MTP, CPM, PLX	Unknown	DAH	Unknown	All three died
Abdelaty et al., 2017	18F	Unknown	VV	Severe ARDS	None	Survived
Pais F et al., 2017	38 F	MTP, PLX	VA	DAH	None	Survived
Castellanos-Moreira et al, 2017	26 F	MTP, CPM	VA	Cardiac shock	None	
Choi et al, 2016	43 F	MTP, IVIG, PLX	VV	DAH	None	Survived
Pacheco et al., 2014	34 F	MTP, CPM	VV	DAH	Multiorgan failure, RRT	Died
	36 M	MTP, CPM	VV	DAH	Thrombosis in circuit, RRT	Survived
Kimura et al., 2015	14 F	MTP, CPM, IVIG, PLX	VV	DAH	None	Survived
Patel et al., 2014	28 F	MTP, CPM, RTX	VV	DAH	RRT	Survived
Leung et al, 2002	24 F	MTP	VA	Cardiac shock	RRT, liver failure	Survived

MTP: methylprednisolone, VA: venoarterial, VV: venovenous, RRT: required renal replacement therapy, CPM: cyclophosphamide, IVIG: intravenous immunoglobulin, DAH: diffuse alveolar hemorrhage, ARDS: acute respiratory distress syndrome.

ECMO is a well-accepted treatment for children with respiratory failure.^21^ An ELSO study showed that the use of extracorporeal life support in children with autoimmune disorders can be successfully supported.^31^ Like their study, most patients in our analysis received ECMO for respiratory support. The decision to initiate ECMO in an SLE patient with pre-existing kidney injury should include thoughtful consideration, as renal dysfunction may complicate management and increase the risk of adverse outcomes. Due to the nature of the registry, we were not able to distinguish if the pre-existing renal dysfunction in our cohort was secondary to SLE or from a different cause.

Decreased survival to hospital discharge in our cohort was associated with cardiac arrest pre-ECMO, need for extracorporeal cardiopulmonary resuscitation, older age and pre-existent bacterial infection before ECMO initiation in adults. Bay et al reported a higher incidence of receiving vasopressors, renal replacement therapy, and experiencing cardiac arrest in non-survivors compared to survivors.^15^ Similarly, we found that adult patients who had cardiovascular, renal, neurologic, and hemorrhagic complications had worse outcomes. None of these complications, however, affected the survival to discharge in our pediatric cohort. Jentzer et al reported from the ELSO registry that delays in ECMO initiation in patients with cardiogenic shock are associated with higher mortality. The authors propose optimization for early ECMO deployment to rescue such patients with cardiogenic shock.^32^ We support that similar consideration should be applied to SLE patients with existing myocardial injury.

Pulmonary hemorrhage in our cohort did not reach statistical difference between survivors versus non-survivors and we were not able to show if pulmonary hemorrhage was associated with decreased survival to discharge. Additionally, SLE patients with significant pulmonary hemorrhage may have not been considered for ECMO at all and not captured in the registry. We recommend that ECMO candidacy in SLE patients with pulmonary hemorrhage should be approached with caution as previously conveyed.^26,33^

Admissions to critical care units of individuals with immune dysfunction have increased globally, as hospital survival rates on these patients have gradually improved over the last decade,^34^ alongside the growing use of awake ECMO strategies that avoids the complications of conventional mechanical ventilation.^35^ The concept of awake ECMO in immunocompromised patients with ARDS had been previously advocated.^35^ A recent Chinese study reported that the strongest predictors of mortality in immunocompromised patients included intubated ECMO and acute kidney injury receiving continuous renal replacement therapy during ECMO.^10^ Tian et al propose that awake ECMO may be achievable for selected immunocompromised patients when no other significant organ dysfunction is present.^10^ In adults, we found that increased survival to discharge was associated with Veno-venous mode (OR 1.71 [1.12-2.6], *p* = .012), and not requiring invasive mechanical ventilation support pre-ECMO (OR 4.57 [1.65-12.67], *p* = .003). A large multicenter study on immunosuppressed patients with severe acute ARDS on ECMO reported a 6-month survival of only 30% and increased mortality associated with longer duration of immunodeficiency status in relation to ECMO cannulation.^36^ Our study did not include information on awake ECMO, long-term survival outcome nor the immunosuppressed status of SLE patients.

This study has several limitations to its retrospective observational design, as well as the constraints of the registry. As a multicenter registry, the dataset likely reflects variability in ECMO indications, patient selection, and management strategies across institutions. The retrospective nature of the study introduces the potential for incomplete or missing data that may have influenced our ability to draw robust conclusions, as well as unmeasured confounders, such as socioeconomic status, baseline comorbidities, or access to care that may affect survival outcomes. Patients with SLE requiring ECMO represent a rare and highly specific population, resulting in a relatively small sample size. The ELSO registry does not include information on SLE disease activity indices. As a result, we were not able to assess whether active disease at the time of ECMO initiation contributed to survival outcomes or if patients with quiescent disease had a different trajectory. Details regarding organ-specific manifestations of SLE were not captured in the dataset. This limited our ability to evaluate the impact of specific organ system dysfunction on the need for ECMO and the impact on survival. Moreover, since it is not known from the current analysis whether the patients that required ECMO had myocardial injury from SLE or from a different cause, a propensity matched analysis with a non-SLE control group could clear up the issue, but unfortunately this is not feasible with the existing database.

The type, intensity, or duration of immunosuppressive therapies, such as corticosteroids, biologics, or conventional disease-modifying antirheumatic drugs remained unknown. This gap prevented analysis of whether immunosuppressive treatment regimens influenced survival and complications. The registry does not provide long-term follow-up or information on functional outcomes, such as quality of life, physical recovery, or progression of SLE following ECMO support. This restricts the ability to evaluate the broader implications of ECMO in this population.

## Conclusion

Given the absence of a significant decrease in survival to hospital discharge within our cohort, ECMO may be considered a viable life-support strategy for pediatric and adult SLE patients. Describing the outcomes of ECMO in SLE in future research is crucial for guiding clinical decision making, risk stratification and prognostication. Identifying factors associated with favorable or worse ECMO outcomes can help improve patient selection, tailor anticoagulation protocols, and refine supportive care strategies. Furthermore, understanding ECMO outcomes can help institutions make evidence-based decisions regarding resource allocation, implement quality improvement interventions focused on reducing complications, and align ethical considerations given the high-risk nature of ECMO.

## Supplemental Material

Supplemental Material - Extracorporeal membrane oxygenation for systemic lupus erythematosus: An ELSO registry analysis Supplemental Material for Extracorporeal membrane oxygenation for systemic lupus erythematosus: An ELSO registry analysis by Maria Pereira, Danielle Guffey, Katherine Doane, Eyal Muscal, Carla Levin, Peter Rycus, Marc Anders and Andrea Ontaneda in Perfusion.
