# Supplementary material for: Extracorporeal membrane oxygenation for systemic lupus erythematosus: An ELSO registry analysis
Source: Perfusion. 2025 May 26;41(2):173–81. doi: 10.1177/02676591251346069 (PMC12916881; doi:10.1177/02676591251346069)
Supplement: Supplemental Material - Extracorporeal membrane oxygenation for systemic lupus erythematosus: An ELSO registry analysis [file sj-pdf-1-prf-10.1177_02676591251346069.pdf]

Appendix

A1. Diagnoses and procedure definitions by ICD 9, ICD 10 and CPT codes

Diagnoses

- Acute kidney injury: 584.9, 584, 583.6, 584.6, 583.7, 584.7, 586, 580.9, 580, S37.0, N17.9, N17.8, N17.0, N17.1, N17.2, N17
- Acute respiratory distress: 518.82, J80
- Anemia: 285.1, 285.9, D62, D64.89, D64.9
- Arrhythmia: 427.0, 427.31, I47.1, I47.2, I48.0, I48.1, I48.2, I48.91, I48.92
- Chronic kidney injury: 585.1, 585.5, 585.2, 585.1, 585.2, 585.4, 585.6, 585.9, N18, N18.1, N18.2, N18.3, N18.30, N18.31, N18.32, N18.4, N18.5, N18.6, N18.9, I12.9, I13.0, Z99.2
- Endocarditis: 424.90, 424.99, I38
- End stage renal disease: 585.6, N18.6
- Glomerulonephritis: 581.1, 581.2, 581.9, 582.1, 582.2, 583.0, 583.1, 583.2, 583.4, 583.9, N002, N003, N004, N005, N007, N00A, N012, N015, N017, N01A, N022, N025, N027, N02A, N032, N033, N034, N035, N037, N042, N043, N044, N045, N047, N04A, N052, N053, N054, N055, N057, N05A, N062, N063, N064, N065, N067, N06A, N07A
- Hemoptysis: 786.30 786.3 784.8, R04.2 R04.1
- Myocarditis: 429.0, I51.4
- Pericardial effusion: 423.9, I09.2, I30.9, I31.3
- Pericarditis: 423.9, I31.9
- Pneumonia: 112.4, 480, 480.9, 482, 482.1, 482.3, 482.41, 483, 484.6, 486, 487, V12.61, A48.1, B25, B37.1, B44, B59, J10.0, J14, J15.0, J15.1, J15.6, J15.9, J16.8, J18, J18.9, J95.851
- Pneumothorax: 512, 512.2, 512.89, B25.0, J90, J93.0, J93.11, J93.81, J93.82, J93.83, J93.9, J95.811
- Pulmonary hemorrhage: 770.3 786.3 786.30 786.31 786.39, R04, R04.81 R04.89, R04.2 R04.9

- Pulmonary hypertension: 416.8, 416.0, I27.20, I27.2, I27.0, I27.29, I27.21, I27.23
- Respiratory failure: 518.81, 518.83, 518.84, 518.52, 518.82, 769, 786.09, J96, J96.0, J96.00, J96.2, J96.91, J96.01, J96.02, RO9.2, J96.20, J96.11, J96.92, J96.12, J96.21, J96.22, J96.02, J96.90, J96.10, J98.9, J99, Z99.11
- Sepsis: 038, 038.9, 995.91, 995.92, A02.1, A41, A41.01, A41.52, A41.9, R65.11, R65.21
- Shock: 785.51, 785.59, R57, R57.1, R57.8, R57.9, R65.21

Procedures:

- Bronchoscopy: 31615, 31622, 31624, 31625, 31633, 31636, 31645, 31646, 31725
- Plasmapheresis: 36513, 36514
- Renal replacement therapy: 90935, 90945, 90947
- Thoracentesis: 32020, 32551, 32555, 32556, 32601
- Tracheostomy: 32020, 32551, 32555, 32556, 32601
